# Supplementary material for: A clinical specific BERT developed using a huge Japanese clinical text corpus
Source: PLoS One. 2021 Nov 9;16(11):e0259763. doi: 10.1371/journal.pone.0259763 (PMC8577751; doi:10.1371/journal.pone.0259763)
Supplement: Attachment — Submitted filename: 07_responce_r1.docx [file pone.0259763.s001.docx]

Thank you for giving us the opportunity to submit a revised draft of the manuscript. We appreciate the time and effort that you dedicated to provide us with your valuable feedback on the manuscript. We are grateful to the reviewers for their insightful comments on our paper. We incorporated changes to reflect most of the suggestions provided by the reviewers.

At first, due to incorrect spelling, we would like to change the title as follows: A clinical specific BERT developed using a huge Japanese clinical text corpus.

Here is a point-by-point response to the reviewers’ comments and concerns.

**# Reviewer1**

1. This work reads very well, and I believe it is an essential contribution to literature as it reduces the shortage of studies with the Japanese language and may trigger other investigations. I have no suggestions on how to improve this work, and I recommend publication in the present form.

**Response to 1**

Thank you for your comment. We trust that the pre-trained model will help research on clinical texts.

**# Reviewer2**

1. The trained BERT model is valuable for Japanese clinical research. However, the differences between the proposed BERT and other general Japanese BERT models in the evaluated task are very small. Although I understand the situation where only a few Japanese evaluation sets in a clinical domain are available, this experimental result is weak for insisting the effectiveness of the clinical-specific Japanese BERT model.

**Response to 1**

Thank you for pointing this out. As a result of the re-experiment, we confirmed that there are no significant differences between the BERTs. In addition, because there are, to our knowledge, few Japanese datasets in the clinical domain to use in our experiments, evaluating the BERT models with an appropriate one was challenging. We modified our results as follows:

**On Page 10,**

Table 4 shows the exact-match accuracies with 95% confidence intervals of four pre-trained BERTs. There were no significant differences among UTH-BERT (0.855), KU-BERT (0.845), and TU-BERT (0.862); however, mBERT significantly showed the lowest accuracy compared to the other BERTs.

Table 4. The exact-match accuracy of each model with five-fold cross validation.

| Model name | Exact match accuracy (95% CI) |
| --- | --- |
| UTH-BERT | 0.855 (0.848 – 0.862) |
| KU-BERT | 0.845 (0.833 – 0.857) |
| TU-BERT | 0.862 (0.857 – 0.866) |
| mBERT | 0.806 (0.794 – 0.817) |

Table 5 shows the label-wise Recall, Precision, and F-measure of each model. There were no significant differences in the mean F-measures among UTH-BERT (0.888), KU-BERT (0.882), TU-BERT (0.888), and mBERT (0.855). The mean F-measure of mBERT tended to be lower than other BERT models, but the difference was not significant. In terms of the performance for each symptom, the mean F values for Flu (0.714) and Fever (0.838) were lower than for the other symptoms.

Table 5. The label-wise performances of each model with five-fold cross validation. The performances shown are Recall, Precision and F-measure.

|  | Flu | Diarrhea | Hay fever | Cough | Headache | Fever | Runny nose | Cold | *Mean F1*  (95% CI) |
| --- | --- | --- | --- | --- | --- | --- | --- | --- | --- |
|  | *Rec. / Prec.*  *F1* | *Rec. / Prec.*  *F1* | *Rec. / Prec.*  *F1* | *Rec. / Prec.*  *F1* | *Rec. / Prec.*  *F1* | *Rec. / Prec.*  *F1* | *Rec. / Prec.*  *F1* | *Rec. / Prec.*  *F1* |  |
| UTH | 0.676/0.858  **0.755** | 0.914/0.919  0.916 | 0.904/0.835  0.865 | 0.928/0.963  **0.945** | 0.947/0.974  **0.960** | 0.797/0.905  0.845 | 0.920/0.927  0.923 | 0.885/0.913  0.898 | 0.888  (0.846 -0.931) |
| KU | 0.594/0.842  0.694 | 0.877/0.956  0.915 | 0.896/0.896  **0.895** | 0.892/0.963  0.926 | 0.947/0.958  0.952 | 0.760/0.927  0.835 | 0.921/0.927  0.924 | 0.890/0.936  **0.912** | 0.882  (0.828-0.935) |
| TU | 0.735/0.692  0.710 | 0.927/0.947  **0.937** | 0.898/0.874  0.885 | 0.916/0.957  0.936 | 0.936/0.982  0.958 | 0.825/0.903  **0.861** | 0.912/0.938  **0.925** | 0.884/0.904  0.893 | 0.888  (0.837-0.939) |
| mBERT | 0.598/0.850  0.696 | 0.867/0.906  0.885 | 0.870/0.822  0.841 | 0.918/0.892  0.905 | 0.928/0.927  0.926 | 0.745/0.890  0.810 | 0.869/0.893  0.879 | 0.902/0.887  0.894 | 0.855  (0.807-0.902) |
| *Mean*  *F1* | 0.714 | 0.913 | 0.872 | 0.928 | 0.949 | 0.838 | 0.913 | 0.899 |  |

2. Although the authors say that "error analysis is required" in L277, improved examples and errors should be presented, and discussion should be made in the paper. These will help readers understand the strength and weakness of the proposed BERT model.

**Response to 2**

Thank you for pointing this out. We agree that an error analysis is important to know the nature of the model. We conducted a qualitative error analysis and described it as follows:

**On Page 12,**

**3.3	Error analysis**

To obtain a better understanding of the UTH-BERT classifier’s mistakes, we qualitatively analyzed its false positive (FP) and false negative (FN) cases in the 640 MedWeb test dataset. The error analysis was conducted for the labels for which UTH-BERT was wrong all five times in the five-fold cross validation. The labels of MedWeb were annotated in terms of three aspects such as Factuality (whether the tweeter has certain symptom or not), Location (whether the symptoms are those of the tweeter or someone in the vicinity or not), and Tense (whether the symptoms exist within 24 hours or not) [19]. Since the MedWeb dataset did not contain information about these perspectives, we manually categorized the error cases based on these aspects. As a result, we obtained eight error types for the FP cases and five error types for the FN cases (Table 6).

Table 6. Interpretations obtained from the results of the error analysis.

| No. | Error | Interpretation | Num. of errors | Example sentence | Incorrect prediction |
| --- | --- | --- | --- | --- | --- |
| 1 | False positive (FP) | FP due to false detection of co-occurring symptoms. | 10 | (ja) インフルかと思って病院行ったけど、検査したら違ったよ。 (en) I thought I had the flu so I went to the doctor, but I got tested and I was wrong. | Fever pos. |
| 2 |  | FP for symptoms mentioned in general topics. | 8 | (ja) 風邪といえば鼻づまりですよね。 (en) To me, a cold means a stuffy nose. | Cold pos.  Runny nose pos. |
| 3 |  | FP for suspected influenza. | 5 | (ja) インフルエンザかもしれないから部活休もうかな。 (en) I might have the flu so I'm thinking I'll skip the club meeting. | Flu pos. |
| 4 |  | FP for fully recovered symptoms. | 5 | (ja) やっと咳と痰が治まった。 (en) My cough and phlegm are finally cured. | Cough pos. |
| 5 |  | FP for metaphorical expressions. | 3 | (ja) 熱をあげているのは嫁と娘だ。 (en) What makes me excited are my wife and daughter. | Fever pos. |
| 6 |  | FP for denied symptoms. | 2 | (ja) 鼻水が止まらないので熱でもあるのかと思ったけど、全然そんなことなかったわ。 (en) My nose won't stop running, which got me wondering if I have a fever, but as it turns out I definitely do not. | Fever pos. |
| 7 |  | FP for symptoms for asking unspecified people. | 2 | (ja) 誰か熱ある人いない？ (en) Anyone have a fever? | Fever pos. |
| 8 |  | FP for past symptoms. | 1 | (ja) ネパールにいったら食べ物があわなくてお腹壊して下痢になった・・・ (en) When I went to Nepal, the food didn't agree with me, and I got an upset stomach and diarrhea... | Diarrhea pos. |
| 9 | False negative (FN) | FN for symptoms that is directly expressed. | 8 | (ja) 痰が止まったとおもったらこんどは頭痛。 (en) Just when I thought the phlegm was over, now I have a headache | Headache neg. |
| 10 |  | FN for symptoms that is indirectly expressed. | 5 | (ja) 中国にいた時は花粉症ならなかったのに再発したー！ (en) Even though I didn't have allergies when I was in China, they're back! | Runny nose neg. |
| 11 |  | FN for symptoms that can be inferred to be positive by being a tweet from a person. | 4 | (ja) 今日花粉少ないとか言ってるやつ花粉症じゃないから。 (en) The people who are saying there's not a lot of pollen today don't have allergies. | Runny nose neg. |
| 12 |  | FN for symptoms that is in the recovery process. | 1 | (ja) インフルが回復してきてだいぶ元気になった！けどあと２日は外出禁止なんだよな。 (en) I've recovered from the flu and feel great! But I'm still not allowed to go out for two days. | Flu neg. |
| 13 |  | FN for symptoms occurring in the tweeter's neighborhood. | 1 | (ja) うちのクラス、集団で下痢事件 (en) There's a diarrhea outbreak in my class | Diarrhea neg. |

1. **FP due to false detection of co-occurring symptoms**

This type of error was categorized as *Factuality*. The example sentence No.1 expresses that the flu is negative, but UTH-BERT incorrectly predicted that the fever and flu are positive. The reason for this error would be that the training data contained sentences in which flu and fever are positive simultaneously. In addition, UTH-BERT could not detect the negative expression of flu, so both flu and fever were incorrectly positive.

2. **FP for symptoms mentioned in general topics**

This was categorized as *Factuality*. The example sentence No. 2 states that a runny nose is a common symptom of a cold. Despite the general topic, UTH-BERT incorrectly predicted that the tweeter has a cold and a runny nose. The reason for this error would be that UTH-BERT failed to distinguish between symptoms that are stated as a general topic and those occurring in a person.

3. **FP for suspected influenza**

This was categorized as *Factuality*. According to the MedWeb annotation criteria, suspected symptoms were treated as positive, but only influenza was treated as negative. (This is because the MedWeb dataset was developed primarily for the surveillance of influenza.) This suggests that difference of annotation criteria between flu and other symptoms, and a lack of sentence expression about suspected flu in the training dataset, led to the errors.

4. **FP for fully recovered symptoms**

This was categorized as *Factuality*. According to the annotation criteria, symptoms are labeled as positive if they are in the recovery process and negative if they are completely cured. In example sentence No. 4, even though the tweeter stated that the cough was cured, UTH-BERT could not recognize *cured* and incorrectly predicted that cough was positive.

5. **FP for metaphorical expressions**

This was categorized as *Factuality*. This error is due to an inability to recognize metaphorical expressions. In example sentence No. 5, the Japanese phrase*熱を上げる* is a metaphorical expression for *excited*, but because it uses the same kanji as *fever*, UTH-BERT incorrectly predicted that fever is positive.

6. **FP for denied symptoms**

This was categorized as *Factuality*. This error is caused by UTH-BERT missing a negative expression.

7. **FP for symptoms for asking unspecified people**

This was categorized as *Location*. Although example sentence No. 7 asks about the presence of fever for an unspecified person, UTH-BERT incorrectly predicted that the tweeter has a fever.

8. **FP for past symptoms**

This was categorized as *Tense*. According to the annotation criteria, past symptoms are treated as negative. This error occurred because UTH-BERT was not able to recognize the tense.

9. **FN for symptoms that is directly expressed**

This was categorized as *Factuality*. Although the sentences directly express that the tweeter has a symptom, this type of errors occurred because UTH-BERT could not detect it.

10. **FN for symptoms that is indirectly expressed**

This was categorized as *Factuality*. This is the type of error that overlooks a symptom that would be inferred to be positive if another symptom was present at the same time. Example sentence No.10 directly expresses that hay fever is positive, but if you have some knowledge, you can guess that runny nose is also positive.

11. **FN for symptoms that can be inferred to be positive because the tweet is from a person.**

This was categorized as *Factuality*. This is also the type of error that overlooks a symptom that is expressed indirectly, but it requires more advanced reasoning. Example sentence No.11 states a general topic, but given that it is a tweet, you can infer that the person has hay fever.

12. **FN for symptoms that is in the recovery process**

This was categorized as *Factuality*. According to the annotation criteria, symptoms are labeled as positive if they are in the recovery process; however, UTH-BERT could not detect it.

13. **FN for symptoms occurring in the tweeter's neighborhood**

This was categorized as *Location*. According to the annotation criteria, if a population in the same space has a symptom, the symptom is annotated as positive regardless of whether the tweeter has the symptom or not. In this case, UTH-BERT predicted it as negative, because there were probably not enough such cases in the training data.

**On Page 18,**

We conducted an error analysis that resulted in 13 different types of error interpretations. Among these interpretations, errors related to the factuality of symptoms were the most common, and errors related to the location and the tense were less common. This bias could be due to the small amount of data labeled for location and tense in the MedWeb dataset, rather than a feature of the UTH-BERT. The most common error type in the FP cases was due to false detection of co-occurring symptoms, and 10 of these errors were found in this analysis. Since the task in the MedWeb dataset is a multi-label classification of eight symptoms, this error would be influenced by co-occurrence relationships of multiple labels appearing in the training dataset. On the other hand, some of the FN error types were found to overlook symptom that were expressed indirectly. A possible way to reduce such oversights would be to prepare many similar cases in the training dataset, but it seemed to be difficult as long as only text was used as a source of information. It was difficult to conduct further analysis since this error analysis was based on manual categorization. For further investigation, it would be possible to apply Shapley additive explanations (SHAP) [20] or local interpretable model-agnostic explanations (LIME) [21] to visualize the effect of the predictions when the input data is perturbed by deleting or replacing input tokens.

Differences in the distribution of words appearing in the clinical text used for pre-training of UTH-BERT and the pseudo-tweet messages used for evaluation may have affected the errors. Clinical texts differ in that they contain objective information about the patient, while pseudo-tweets message contain subjective information about the tweeter. Another difference is that the former is written in a literary style, while the latter is written in a spoken form. Because of these differences, there may be cases where the representation of the UTH-BERT pre-acquired tokens were not maximally utilized in the pseudo-tweet messages, leading to errors. A limitation of our error analysis is that it was not possible to compare the error trends between the BERT models because we did not perform the error analysis for KU-BERT and TU-BERT. Moreover, given that our developed BERT was evaluated exclusively on the NTCIR-13 MedWeb task, there is currently a limitation in the generalizability of the performance.

3. The comparison between BERT and other classical machine learning methods such as SVM and LR does not make sense in this paper because the superiority of BERT models has been shown in many papers. Furthermore, SVM with KU or TU vocabularies does not make sense because these (subword) vocabularies are not for SVM nor LR.

**Response to 3**

As you commented, the superiority of BERT models has been shown in previous studies. We removed the sentences that mentioned SVM and LR.

4. There are many typos and some misunderstandings for BERT. Please check the followings carefully.

**Response to 4**

Thank you for your careful attention. We revised our manuscript based on your comments, and the manuscript was subsequently proofread by native English speakers. We believe it is significantly improved from the previous submission.

5. "A subword that starts with "##" represents a subword that is not the beginning of a word."

**Response to 5**

Thanks for the important comment. We modified the manuscript as follows:

**On Page 4,**

A subword that starts with ## represents a subword that is an appendage to another word.

6. - L122: What does "the sequence" mean? It means a word sequence? The word2vec embeddings, for example, are trained from a word sequence, and so this explanation is inaccurate for mentioning the difference between BERT and existing embeddings.

**Response to 6**

We modified the manuscript to clearly distinguish between BERT and word2vec as follows:

**On Page 6,**

The novelty of BERT is that it took the idea of learning word embeddings one step further, by learning each embedding vector considering the co-occurrence of words.

7. - L123: I think the clause "which learns sentence expressions .. between words" is misunderstanding. The self-attention in BERT can learn token embeddings as well as sentence embeddings.

**Response to 7**

We modified the manuscript as follows:

**On Page 6,**

To do this, BERT utilizes the self-attention mechanism, which learns sentence and word embeddings by capturing co-occurrence relationships between those embeddings.

8. - L132: I can't understand the sentence "more appropriate representation of sentences is obtained".

**Response to 8**

As you commented, our description was inappropriate. We revised the manuscript based on the original BERT paper (Devlin et al. 2018) as follows:

**On Page 6,**

This task masks, randomly replaces, or keeps each input token with a certain probability, and estimates the original tokens. Estimating not only the masked tokens but also the replaced or kept tokens help to keep a distributional contextual representation of every input token.

9. - L142: I think the verb "pinch" is not appropriate in this context
10. - L143: I think there is a misunderstanding in the NSP task. For a sentence in a document, a random sentence as a negative example is chosen from other documents. Therefore, it is a usual way that all the sentences in a document in one day are regarded as one document. The explanation from L142 to L147 does not make sense.

**Response to 9, 10**

Thank you for pointing this out. In response to your suggestion, we deleted the inappropriate expressions and revised the text as follows:

**On Page 7,**

To develop such a training dataset, for two consecutive sentences in the original dataset, the first sentence is connected to the original second sentence with a probability of 50% as a positive example. The remaining 50% of the time, the first sentence is connected to a randomly sampled sentence as negative example. We treated all sentences appearing in a document recorded in one day for a patient as consecutive sentences.
